# Real World Study to Evaluate the Effectiveness of Varenicline and Cognitive-Behavioural Interventions for Smoking Cessation

**DOI:** 10.3390/ijerph6041530

**Published:** 2009-04-21

**Authors:** Josep Ma. Ramon, Eugeni Bruguera

**Affiliations:** 1 Smoking Cessation Clinic. Hospital Universitario de Bellvitge. Feixa Llarga s/n 08907 Hospitalet. Barcelona, Spain; 2 Smoking Cessation Clinic. Hospital Universitari de la Vall d’Hebron. Passeig de la Vall d’Hebron 119-129. 08035 Barcelona, Spain; E-Mail: eugeni.bruguera@gmail.com

**Keywords:** Smoking cessation, varenicline, cognitive-behavioural intervention, continuous abstinence

## Abstract

A prospective pragmatic interventional study was conducted to evaluate the effectiveness of varenicline for smoking cessation among a cohort of motivated smokers attending two smoking cessation clinics. Smokers between 18 and 65 years who had smoked 10 or more cigarettes per day were included. All participants received cognitive-behavioural varenicline according to approved dose and prescriptions. Continuous abstinence, validated by exhaled CO levels, was assessed in each control. A total of 264 smokers – 155 males (58.7%) and 109 females (41.3%) – were included. Mean age was 43.7, amount smoked was 23 cigarettes per day and 61.4% had at least one prior attempt to quit. The continuous abstinence rate at end of treatment (12 wks) was 58.3%. Conclusions: varenicline and cognitive-behavioural intervention are effective for smoking cessation with high continuous abstinence rates when are used in a clinical setting.

## 1. Introduction

Cigarette smoking continues to be the major cause of morbidity and mortality in Western populations. Several studies [1,2] reported that 60–70 percent of smokers want to quit and intend to stop, but only 3–5% of them remain abstinent for a year [3]. Smoking is a complex behaviour influenced by both genetic and environmental factors [4,5]. Nicotine is the component of tobacco that is responsible for reward, reinforcement and the withdrawal effects of cigarettes [5]. When a smoker inhales cigarette smoke, nicotine diffuses rapidly into the brain where it binds to nicotinic cholinergic receptors (nAChRs). One physiological effect of nAChR activation is dopamine release in the *nucleo acumbens*, an important focus for the reward properties of nicotine [6].

Currently available first line smoking cessation therapies include nicotine replacement therapy, bupropion and, most recently, varenicline. Nicotine replacement therapy acts on nAChRs to replace the effects of nicotine from tobacco and the principal action is the relief of withdrawal symptoms and, a second mechanism, is positive reinforcement, particularly, for the arousal and stress relieving effects [6]. The precise mechanism of bupropion for smoking cessation is unclear; however its effects may be related to reduced cravings and withdrawal symptoms and, on the other hand, prevention of dopamine reuptake [7].

Varenicline, licensed by the European Agency for the Evaluation of Medical Products in September 2006 and introduced for clinical use in Spain in January 2007, is a selective α4β2 nAChR partial agonist developed for smoking cessation. The agonist activity reduces craving and withdrawal symptoms and their antagonist properties, on the other hand, it reduces the reward experienced by those who relapse during treatment increasing the abstinence rates [8,9]. Several randomized, double-blind placebo controlled trials [10–14] had reported significantly higher abstinence rates by varenicline compared to bupropion, NRT and placebo. These clinical trials provide scientific evidence of the efficacy and safety of varenicline used as smoking cessation therapy however; the final evaluation of clinical effectiveness is when varenicline is used in real-life settings and no studies have been published that report data on predictors of success when varenicline is used for smoking in such a real-world setting.

The aim of this study was to assess the effectiveness of varenicline and cognitive-behavioural intervention when both are used under real clinical practice conditions during a 12 weeks period of treatment and investigated the role of different factors as predictors of abstinence at the end of treatment with varenicline.

## 2. Methods

### 2.1. Study Design

A prospective pragmatic interventional study was carried out among motivated to quit smokers attending two smoking cessation clinics in Barcelona, Spain as routine cases between January and July 2007. The study protocol was approved by the ethical review committee of our institution.

### 2.2. Participants

Participants were chain smokers motivated to quit and attending two specialist’s smoking cessation clinics during the study period. Smokers between the ages of 18 and 65 years and who had smoked an average of 10 or more cigarettes per day during the previous month were included in the study. Twenty eight smokers (9.0%) with current depressive disorder, a previous psychosis or bipolar disorder, pregnancy or breastfeeding were excluded from the study. Psychiatric comorbidity was assessed using BDI inventory and brief psychiatric rating scale. Thirty percent of smokers (29.9%) had a major chronic associated disease including cardiovascular disease, diabetes, chronic bronchitis and asthma.

### 2.3. Intervention

All smokers received a standardized program for smoking cessation including pharmacological and cognitive-behavioural interventions (developing a specific action plan and alternative behaviours). Subjects received five 20–30 minute individual consultations during the treatment period and varenicline was prescribed according to indications, 0.5 mg/day for 3 days, 0.5 mg twice daily and 1 mg twice daily for 11 weeks. The pharmacologic treatment begins always one week before the target quit day. Smokers attended follow-up control visits at weeks 2,4,6,8 and 12 after quit day.

### 2.4. Assessments

At baseline a detailed smoking history, health data and participant’s motivation, using Richmond test, was gathered. Questionnaires included questions as sex, age, when cigarette use started, number of cigarettes per day and Fagerström test for nicotine dependence (FNTD), previous attempts to quit, presence of other smokers in the household and former and current diseases and medications. Self-reported cigarette consumption, exhaled CO level, weight and possible adverse effects were measured at all follow-up visits. Adverse event were assessed systematically in each control using a structured questionnaire. Effectiveness was measured as continuous abstinence rate (CAR) confirmed by exhaled CO levels of 10 ppm or below from week 2 through week 12. On the other hand, point prevalence of abstinence was defined as no smoking in the previous seven days as verified by an expired level of CO lower than 10 ppm.

### 2.5. Statistical Analysis

Data were first entered into a relational database (Access 2000, Microsoft Corp, Redmond, Washington) and the converted into a SPSS file (SPSS 12.0, Chicago, Illinois). In the descriptive analysis, categorical variables are expressed as proportions and continuous variables as mean (standard deviation). Univariate analysis of variables from the entire population was performed using chi square test and two-sample test for continuous data. All test using a significant level of 0.05.

Multivariate logistic regression was used to identify independent predictors (age, gender, number of cigarettes per day, number of attempts, FTND score and associated disease) associated with continuous abstinence at week 12 as primary outcome. Models were fitted including all variables associated with the outcome. Any variable was subsequently eliminated from the model if the chi-square statistic of likelihood ratio test indicated no statistical significance. No substantial confounding of odds ratio estimates for the other variables were observed. Possible effect modification was analyzed by fitting interaction terms between variables and resulting models were compared by likelihood ratio test. Results are showed as crude and adjusted odds ratios and their 95% confidence intervals. Subjects who did not attend follow-up visits were considered smokers.

## 3. Results

Baseline information and demographic characteristics of the participants are reported in Table 1. Of the 264 included smokers, 155 (58.7%) were males and 109 (41.3%) females, with an average age of 43.7 years who smoked an average of 23 cigarettes per day and 61.4% had at least one prior quit attempt. Forty percent of smokers indicated other smokers in the household and 29.9% of included subjects had an associated disease. The drop-out rate of consultation attending was 37.8% (n = 71) without statistical differences by sex and age (p > 0.05).

After 12 weeks of follow-up, 154 of the 264 smokers were sustained abstainers post quit-day with an overall success rate of 58.3% (Table 2). When differences of continuous abstinence were assessed by gender, males and females showed similar rates after 4 and 6 weeks of follow-up (Table 2). At week 12, female continuous abstinence rate (61.5%) was higher than for males (56.1%), although the difference was not statistically significant (OR 0.91; p 0.06).

The continuous abstinence rate for the last four weeks of treatment (weeks 9–12) was 61.3% (162/264). When the successful-12 week predictors were assessed in a univariate model (Table 3), the number of cigarettes consumed per day, and FTND score, were clear predictors of abstinence at the end of treatment (OR 0.58, 95% CI 0.2–0.7 and OR 0.71, 95% CI 0.7–0.9, respectively). On the other hand, number of previous attempts (OR 0.79, 95% CI 0.2–1.0) and presence of other smoker in the household (OR 0.59, 95% CI 0.2–0.9) were also associated with continuous abstinence at 12 week (Table 3). The effect of associated disease was not significant (Table 3).

When all significant predictor factors were included in a multivariate model (Table 3), estimated OR adjusted by age, gender, clinic and all variable in the table, nothing changed significantly. Smoking less than 30 cigarettes per day, one or more previous attempts and no presence of other smokers in the household persisted as significant predictors of smoking cessation after 12 weeks of treatment.

**Figure 1.**
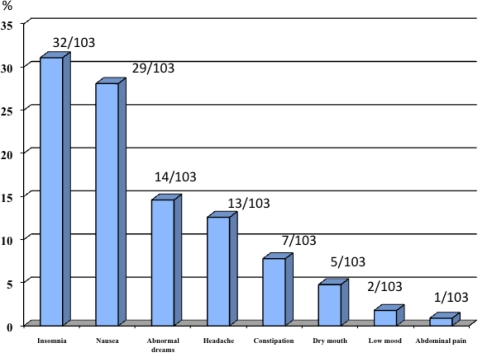
Percentage of adverse events. N = 103.

Finally, 39.1% (103/264) of all smokers showed any adverse event observed mostly during the first six weeks of treatment. Insomnia was the most frequent side effect with 31.1% of smokers. Nausea occurred in 29 smokers (28.7%) and of those, it was of low/mild intensity in 81% of cases (Figure 1). Other common side effects were abnormal dreams (13.6%), headache (12.6%) and constipation (7.8%).

Less than 2% of smokers without previous history of psychiatric disease reported low mood or clinical depression. Varenicline was discontinued in nine cases, one of them due to a depressive episode and in eight smokers (3%) due to side effects such as nausea (5/8) and insomnia (3/8). All of them were excluded from the analysis.

## 4. Discussion

Previous trials [10–14] had reported that varenicline was most effective than placebo, nicotine patches and bupropion for smoking cessation. In a recent meta-analysis [15], where data from three trials of varenicline with an active bupropion arm was used, varenicline was superior to bupropion with an estimated OR of 2.18 (95% CI 1.09–4.08). The present study show data on abstinence after the standard period of clinical treatment for smoking cessation with varenicline and cognitive-behavioural intervention and, also, defined determinants of success in a clinical context.

The effectiveness data presented in this study are similar to the results reported in the original clinical trials for varenicline. The corresponding real-life effectiveness and continuous abstinence at 12 weeks estimated ranged from 56.1% for males to 61.5 for females. The week 9–12 continuous abstinence rate among our cohort of smokers was 61.3%, comparable with the results reported by Aubin *et al.* (55.9%) [14], but higher than that reported for varenicline group by Jorenby *et al.* (43.9%) [11] and Gonzales *et al.* (44.0%) [12].

Identification of individual characteristics that predict success in smoking cessation is highly desirable and a number of common predictors have been identified as personal, social and psychopathological factors [16]. Only a study by Stapleton *at al*. [17] has reported results on predictor factors in smoking cessation when varenicline has been used as pharmacological treatment. In our study, age, other smokers in household, previous attempts and number of cigarettes emerged as predictors of smoking cessation for the smokers who use varenicline as pharmacological treatment.

Different studies [16] have shown that age is a determinant of cessation in the general population and, a better success rate has always been reported among older smokers. Stalpleton *et al.* [17] reported that age and consumption of cigarettes are prognostic factors for cessation in a cohort who received varenicline. Our results suggest that smokers who smoked less than 30 cigarettes per day had higher success rates, independently of other factors such as FTND score, age, gender and previous quit attempts, similar to the data reported by Stapelton *et al.* [17]. Finally, cohabiting with non-smokers and number of previous attempts were also associated with success.

On the other hand, the results confirm previously data from clinical trials that side effects are common during treatment with varenicline although moderate in severity and, in our study, only a 3% of smokers need to stop varenicline due to side effects. Although insomnia has been observed more frequently than previous studies it could be explained by different reasons than the varenicline use but, in general, reported side effects in our study, when vareninline is used in real world setting, are similar those observed previously.

The major limitation of the study is the inclusion of smokers who received expert and behavioural support for smoking cessation and observed rates may be different when varenicline could be used as pharmacological support in brief counselling and, on the other hand, the absence of a control group without behavioural intervention.

In conclusion, varenicline associated to behavioural intervention are effective for smoking cessation, when effectiveness is measured at the end of treatment period, with high continuous abstinence rates, even more studies with control group without behavioural and using a placebo group are needed in the future.

## Figures and Tables

**Table 1.** Demographic and baseline characteristics of included smokers by gender.

	**Male N = 155**	**Female N = 109**	**Total N = 264**	**p value**
Age mean (SD)	45.9 (10.4)	39.5 (7.9)	43.7 (10.1)	< 0.001*
Cig/day. Mean (Range)	28 (15–50)	22 (10–40)	23 (10–50)	0.008*
N° previous attempts. Mean (SD)	1.6 (1.0)	1.4 (1.3)	1.5 (1.1)	0.6*
Age starting smoking. Mean (SD)	17.1 (5.0)	17.5 (3.4)	17.3 (4.5)	0.9*
Fagerström test score. Mean (SD)	6.4 (2.5)	6.0 (2.6)	6.3 (2.5)	0.4*
Longest previous abstinence (days) Mean (Range)	6.2 (0–60)	7.3 (0–100)	6.5 (0–100)	0.7*

* p value for T-test.

**Table 2.** Continuous abstinence rate by gender.

	**wks 2–4 CAR (n/N)**	**wks 2–6 CAR (n/N)**	**wks 2–12 CAR (n/N)**
Males	79.3% (123/155)	67.1% (104/155)	56.1% (87/155)
Females	77.9% (85/109)	66.0% (72/109)	page 6 second parag.
Total	78.8% (208/264)	66.7% (176/264)	58.3% (154/264)

CAR: Continuous abstinence rate.

**Table 3.** Estimated crude Odds Ratios for variables associated with continuous abstinence.

	**Abstainers at week 12 (Quitters/Total)**	Crude OR for abstinence at week 12 (95% CI**)	Adjusted OR* for abstinence at week 12 (95% CI**)
Associated Disease			
None	114/185	1.0	
Yes	40/79	1.21 (0.81–1.93)	
Cig/day			
> 30	25/68	1.0	1.0
21–30	41/58	0.52 (0.16–0.60)	0.46 (0.10–0.68)
≤ 20	88/138	0.58 (0.20–0.78)	0.50 (0.16–0.80)
			p for trend 0.02
Previous attempts			
0	51/102	1.0	1.0
>= 1	103/162	0.79 (0.25–1.00)	0.67 (0.22–0.96)
FTND score			
> 6	70/136	1.0	1.0
3–6	49/80	0.84 (0.47–1.97)	0.74 (0.38–2.0)
< 3	35/48	0.71 (0.17–0.90)	0.80 (0.51–1.8)
			p for trend 0.4
Other smoker in household			
Yes	43/105	1.0	1.0
Non	111/159	0.59 (0.25–0.98)	0.50 (0.21–0.87)

OR : Odds Ratio;

* Adjusted Odds Ratios by age, gender, center and all variable in the table;

** 95% CI : 95% confidence intervals.
